# Elevated TIM3 expression on bone marrow T cells drives immune dysfunction in early relapsed blood cancer after allogeneic hematopoietic stem cell transplantation

**DOI:** 10.1186/s40164-025-00697-6

**Published:** 2025-08-14

**Authors:** Thi Thuy Duong Pham, Su-Young Choi, Jeong Suk Koh, Bu-Yeon Heo, Sang-Woo Lee, Myung-Won Lee, Wonhyoung Seo, Yunseon Jang, Jung-Hyun Park, Deog-Yeon Jo, Seungyeul Yoo, Jaeyul Kwon, Ik-Chan Song

**Affiliations:** 1 Department of Medical Science, College of Medicine, Chungnam National University, Daejeon, South Korea; 2 Brain Korea 21 FOUR Project, College of Medicine, Chungnam National University, Daejeon, South Korea; 3 Department of Internal Medicine, College of Medicine, Chungnam National University, Daejeon, South Korea; 4 Translational Immunology Institute for Medical Science, College of Medicine, Chungnam National University, Daejeon, South Korea; 5 Department of Genetics and Genomics Science, Icahn School of Medicine at Mount Sinai, NY New York, USA

**Keywords:** T cell immunoglobulin and mucin domain 3 (TIM3), Inhibitor receptors, Allogeneic hematologic stem cell transplantation (HSCT), Early relapse (ER), Double negative t cells (DNTs), Regulatory t cells (Treg), Sabatolimab, Leukemia immune microenvironment

## Abstract

Relapse after allogeneic hematopoietic stem cell transplantation (allo-HSCT) remains the leading cause of treatment failure in patients with hematologic malignancies. To better understand the mechanisms underlying early relapse (ER), we comprehensively explored the expression of inhibitory receptors (IRs) in bone marrow (BM) T cells at differentiation stage and examined transcriptional differences. Among the evaluated IRs, TIM3 was significantly upregulated in CD3^+^T cells of patients with ER compared to patients with complete remission (CR). Notably, double-negative T (DNT) cells, a unique subset with MHC-independent cytotoxic potential, constituted a high proportion of BM T cells and expressed increased TIM3 expression in ER patients. Moreover, regulatory T cells (Tregs) showed elevated TIM3 levels, contributing to an immunosuppressive microenvironment after allo-HSCT. Transcriptomic analysis revealed downregulation of cytotoxic granules and effector genes in DNT cells from ER patients. Functional assays demonstrated that TIM3 blockade with sabatolimab restored T cell cytotoxicity, leading to enhanced leukemia cell apoptosis in ER patients. These findings highlight TIM3 as a critical regulator of T cell exhaustion and immune suppression in patients with ER and provide a rationale for the therapeutic use of TIM3 blockade in preventing and treating relapses after allo-HSCT.

**Supplementary Information:**

The online version contains supplementary material available at 10.1186/s40164-025-00697-6.

To the editor,

Allogeneic hematopoietic stem cell transplantation (allo-HSCT) remains a curative option for hematologic malignancies, primarily through the graft-versus-leukemia effect [1]. However, approximately one-third of patients relapse following transplantation, with particularly poor outcomes observed in early relapse (ER) cases [2]. Currently, there are no established therapies that effectively reverse T cell exhaustion in these patients [3]. Although immune checkpoint inhibitors targeting PD1 or CTLA4 have been evaluated, their clinical efficacy in relapsed post-HSCT settings has been limited [4, 5]. Among immune inhibitory receptors (IRs), T cell immunoglobulin and mucin-domain containing-3 (TIM3) has emerged as a promising therapeutic target [6]. TIM3, expressed on T cells, impairs cytotoxic CD8^+^T cell responses and supports leukemic stem cell persistence in myeloid malignancies [7]. And, double negative T (DNT) cells represent a unique subset of T cells expressing CD3 and αβ^+^ or γδ^+^ T cell receptor (TCR) while lacking CD4, CD8, and CD56 expression [8]. DNT cells can kill malignant cells via major histocompatibility complex (MHC)-independent mechanisms and have shown therapeutic potential in relapsed or refractory AML patients [9, 10]. Recently, adoptive cell therapy using DNTs has been explored as a novel treatment approach in cancer immunotherapy [11]. Here, we systematically analyzed the expression of IRs on bone marrow (BM) T cells at different differentiation stages and performed single-cell transcriptomic profiling to investigate transcriptional alterations associated with ER in a cohort of 74 allo-HSCT recipients (Table S1).

Flow cytometry (gating strategy in Figure 1S) revealed a marked reduction in CD3^+^ and CD8^+^T cells in ER patients compared to those in complete remission (CR), while CD4^+^T cell levels remained similarly low in both groups (Fig. 1A-C). Notably, the proportion of DNT cells was significantly increased in ER patients (Fig. 1D). Among the inhibitory receptors (IRs) analyzed—TIM3, TIGIT, CTLA4, PD1, and LAG3—TIM3 showed the highest expression in CD3^+^T cells, with elevated levels observed specifically in the DNT subset of ER patients (Fig. 1E-F). However, there was no significant difference in the expression of TIM3 in CD4^+^T and CD8^+^T cells between the two groups (Figure S2). This was further confirmed by uniform manifold approximation and projection analysis, which showed that TIM3 expression was higher in the DNT cluster in ER patients (Fig. 1G). Furthermore, TIM3 expression was elevated in the effector memory re-expressing CD45RA (TEMRA) subset of CD8^+^T cells in ER patients (Figure S3). These findings suggest that effector T cells in ER patients display impaired cytotoxic function, associated with increased TIM3 expression, particularly in the DNT subset. Additionally, the expression of CD95 (Fas/apoptosis antigen 1), an important receptor mediating cell death [12], was significantly reduced in T cells from the ER group (Figure S4). These results further confirm that T cells in ER patients have reduced ability to eliminate leukemia cells.

**Fig. 1:**
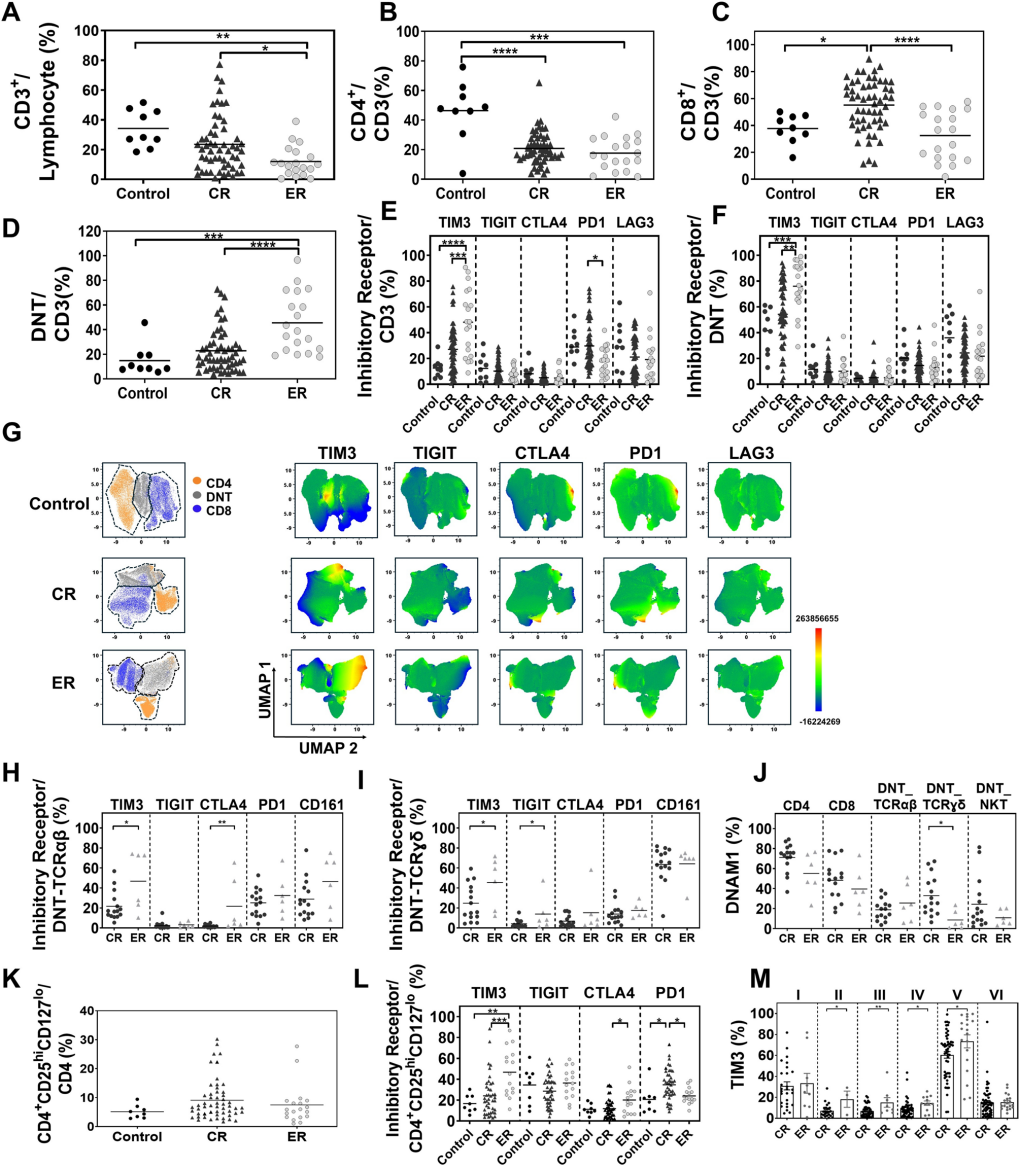
T-cell subsets and the expression level of the inhibitory receptors on the BM T cells. **A** Proportion of CD3^+^T cells among total lymphocytes in the control (*N* = 9), CR (*N* = 55) and ER (*N* = 19) groups. **B** Percentage of CD4^+^T cells among the total CD3^+^T cells. **C** Percentage of CD8^+^T cells among the total CD3^+^T cells. **D** Percentage of DNT cells among the total CD3^+^T cells. **E** Percentages of IR-positive cells in total CD3^+^T cells. **F** Percentages of IR-positive cells in DNT cells. **G** Uniform manifold approximation and projection (UMAP) visualization showing expression levels of each IR on CD3^+^T cells in each group. **H** Proportion of IR-positive cells within the DNT-TCRαβ subset in CR (*N* = 15) and ER (*N* = 6) groups. **I** Proportion of IR-positive cells within the DNT-TCRɣδ subset. **J** Ratio of DNAM1 expresssion across CD3^+^T cell subtypes (CD4^+^T cells, CD8^+^T cells, DNT cell subtypes including DNT-TCRαβ, DNT-TCRɣδ and DNT-NKT). **K** Percentage of conventional (CD4^+^CD25^hi^CD127^lo^) Tregs among CD4^+^T cells in the control (*N* = 9), CR (*N* = 55) and ER (*N* = 19) groups. **L** Proportion of IR-positive cells among the conventional (CD4^+^CD25^hi^CD127^lo^) Tregs. **M** TIM3 expression level in Tregs subsets between CR and ER groups

To further characterize the heterogeneity of DNT cells, including TCRαβ, TCRγδ, and natural killer T (NKT) cell features [13], we performed additional immunophenotyping (Figure S5). No significant differences were detected in the ratios of DNT subsets, including TCRαβ^+^ and TCRγδ^+^ DNT as well as NKT-DNT, between the two groups (Figure S6). TIM3 expression was significantly higher in both DNT-TCRαβ and DNT-TCRγδ subsets in the ER patients (Fig. 1H-I). Given that DNT-medicated cytotoxicity relies on DNAM1 in leukemic cells [14], we found that DNAM1 was reduced in DNT-TCRγδ subset in the ER patients (Fig. 1J). These findings implicate impaired cytotoxicity of DNT cells in ER, linked to high TIM3 and low DNAM1 expression.

We also investigated regulatory T cells (Tregs), subclassifying them into naïve, active, and non-suppressive subsets (Figure S7) [15]. While overall Treg frequencies did not differ between the two groups (Fig. 1K), TIM3 expression was elevated in total and active Tregs in ER patients (Fig. 1L–M). Given that TIM3 enhances Treg suppressive function via IL-10 secretion [16], these findings suggest that increased immunosuppressive activity by Tregs may contribute to immune dysfunction in ER.

To further dissect these immune dysfunctions, we performed single-cell RNA sequencing on BM T cells from two CR and two ER patients (Table S2 & Figure S8). Flow cytometry confirmed increased TIM3 expression in CD3^+^T cells and DNT cells in the ER cohort (Figure S9). Although the disease types differed between the CR and ER groups, there was no difference in TIM3 expression between disease types (Figure S10). And TIM3 expression in myeloid-derived suppressor cells was not differ between the ER and CR groups (Figure S11). Transcriptomic analysis revealed downregulation of cytotoxic granules (*PRF1*,* GZMA*,* GZMB*) and effector genes (*CD226*,* TRDC*) in DNT cells from ER patients (Fig. 2A). Among these, *CD226* (also known as DNAM1) plays a key role in mediating cytotoxicity in DNT cells, while *TRDC* contributes to the cytotoxic function of γδ^+^DNT cells via the Fas/FasL signaling pathway [17]. Furthermore, *IKZF2* expression was lower in the ER group than in the CR group, and *IKZF2* is also associated with the cytotoxic function of DNT cells (Fig. 2A) [18].

**Fig. 2:**
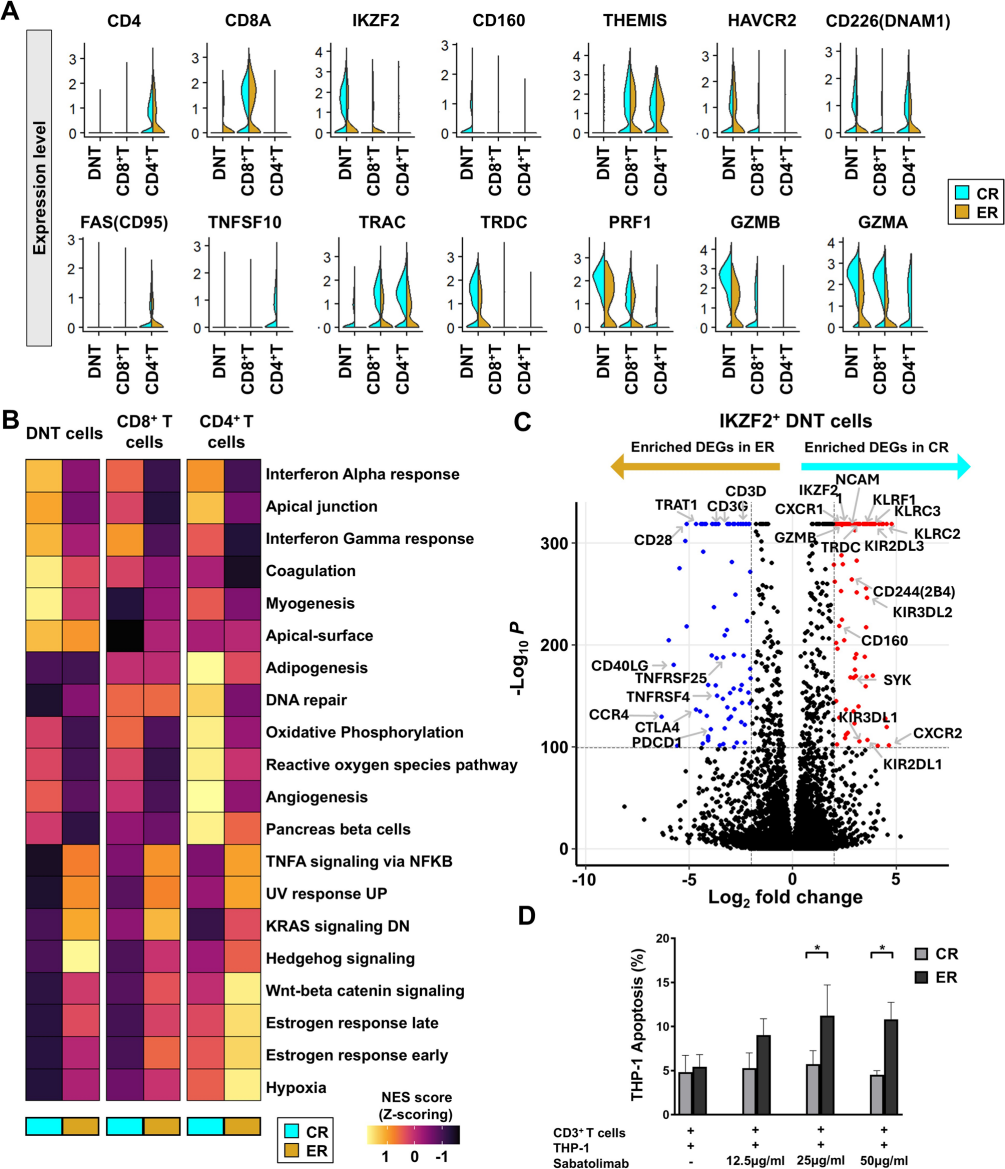
Transcriptional landscape of T-cell subsets and cytotoxicity assay with TIM3 blockade. **A** Violin plot showing expression levels of representative genes (*CD4*,* CD8A*,* IKZF2*,* CD160*,* THEMIS*,* HAVCR2*,* CD226*,* PRF1*,* GZMB*,* GZMA*) in the DNT, CD4^+^ and CD8^+^T cell subsets. **B** Single-sample gene set enrichment analysis (ssGSEA) comparing DNT, CD4^+^ and CD8^+^T cells. **C** Volcano plot illustrates differentially expressed genes between complete remission (CR) and early relapse (ER) groups, with 321 genes upregulated in CR and 315 genes upregulated in ER. **D** T-cell cytotoxicity assay against THP-1 cells following TIM-3 blockade using sabatolimab

Gene set enrichment analysis revealed suppression of oxidative phosphorylation, reactive oxygen species, and interferon responses in ER T cells, indicating impaired immune function (Fig. 2B). Additionally, DNT cells in ER exhibited upregulation of exhaustion markers including *PDCD1* and *CTLA4* (Fig. 2C), supporting a phenotype of functional exhaustion. Conversely, DNT cells from CR patients demonstrated an upregulation of NK cell-associated markers, including *KIR*, *KLRCs*, *KLRF1*, and *CD160*, suggesting potential activation of these cells in a manner resembling NK cells. Additionally, the CR group exhibited an upregulation of *IKZF2*, which is indicative of preserved cytotoxic function (Fig. 2C).

Based on these findings, we hypothesized that TIM3 blockade could restore T cell cytotoxicity. A cytotoxicity assay using sabatolimab (anti-TIM3 antibody) and BM-derived T cells from ER patients showed significantly enhanced apoptosis of THP-1 leukemia cells (Fig. 2D), indicating restored T cell cytotoxic function. In CR patients, despite low TIM-3 expression, T cells exhibited limited cytotoxicity against AML cell lines due to insufficient priming and a metabolically quiescent state (Fig. 2B and D). Meanwhile, TIM3 blockade is more effective in leukemia cells that highly express TIM3 ligands such as galectin-9 or CEACAM1 [19]. The THP-1 cell line used in our study overexpresses FLT3 and secretes galectin-9 at high levels, providing a relevant model for applying TIM3 blockade.

In summary, our data reveal that early post-transplant relapse is associated with TIM3-mediated immune dysfunction in both DNT and Treg populations. Elevated TIM3 expression contributes to impaired T cell cytotoxicity and increased immunosuppressive tumor microenvironment. Importantly, the TIM3 blockade restored cytotoxic function in vitro, suggesting a promising therapeutic approach for patients experiencing early relapse after allo-HSCT.

## Supplementary Information

Below is the link to the electronic supplementary material.

Supplementary Material 1

Supplementary Material 2

Supplementary Material 3

Supplementary Material 4

Supplementary Material 5

Supplementary Material 6

Supplementary Material 7

Supplementary Material 8

Supplementary Material 9

Supplementary Material 10

Supplementary Material 11

Supplementary Material 12

Supplementary Material 13

Supplementary Material 14

Supplementary Material 15

Supplementary Material 16

## Data Availability

Original data can be requested from the corresponding author (petrosong@cnu.ac.kr) upon IRB approval and a data transfer agreement. The scRNAseq raw data generated in this study have been submitted in the Gene Expression Omnibus database with accession code GSE292910. The code used for this data analysis is stored in the Git-Hub repository and can be accessed and used at https://github.com/Syoung-Choi/CNU_Leukemia_Pj1.

## Abbreviations

Allo-HSCT: Allogeneic hematopoietic stem cell transplantation
ER: Early relapse
CR: Complete remission
TIM3: T cell immunoglobulin and mucin-domain containing-3
IRs: Inhibitory receptors
BM: Bone marrow
DNT: Double negative T
NKT: Natural killer-T
Tregs: Regulatory T cells
UMAP: Uniform manifold approximation and projection
ssGSEA: Single-sample gene set enrichment analysis
TEMRA: Effector memory re-expressing CD45RA
