# Supplementary material for: Elevated TIM3 expression on bone marrow T cells drives immune dysfunction in early relapsed blood cancer after allogeneic hematopoietic stem cell transplantation
Source: Exp Hematol Oncol. 2025 Aug 14;14:107. doi: 10.1186/s40164-025-00697-6 (PMC12355862; doi:10.1186/s40164-025-00697-6)
Supplement: Supplementary file 1 — Supplementary Material 1 [file 40164_2025_697_MOESM1_ESM.docx]

**Methods**

**Study design and patient characteristics**

We retrospectively analyzed consecutive adult patients (age > 18 years) with hematological malignancies who underwent allo-HSCT at Chungnam National University Hospital (Daejeon, South Korea) between April 2021 and October 2024. Patients with second transplantation or refractory disease were excluded. Of the 74 total patients enrolled, 33 were male and 41 were female. All the patients underwent mobilized peripheral blood stem cell transplantation (Table S1). All patients received rabbit ATG (thymoglobulin, 1.5 mg/kg; Sanofi-Aventis, Paris, France) from day 1 to 3 for GVHD prophylaxis. Post-transplant cyclophosphamide (PTCy, 50 mg/kg) was administered on days +3 and +4 to patients who underwent haplo-identical HSCT. Normal bone marrow samples were obtained from healthy bone marrow transplantation donors. Early relapses were defined as a morphological relapse within 6 months after allo-HSCT. Acute GVHD was graded using the Mount Sinai Acute GVHD International Consortium (MAGIC) criteria.

**Cell Staining and flow cytometry.**

Frozen BM samples were thawed and cultured overnight at 37°C in complete RPMI medium supplemented with 10% fetal bovine serum (FBS; 10082147, Gibco, ThermoFisher, USA) and 1% penicillin/streptomycin at a concentration of 1–2 × 10⁶ cells/mL. Cell viability was assessed using LIVE/DEAD Fixable Violet dye (L34955, ThermoFisher Scientific, USA). Cells were stained with fluorochrome-conjugated monoclonal antibodies (listed in Table S3), incubated for 30 minutes at 4°C, and washed with phosphate-buffered saline (PBS) containing 20% bovine serum albumin (BSA) and 0.1% sodium azide. Data acquisition was performed on a BD LSRFortessa™ X-20 flow cytometer (BD Biosciences, USA), and data were analyzed using FlowJo™ Software v10.10.0 (BD Biosciences, RRID:SCR_008520).

Flow cytometry revealed a trend toward increased TIM3 expression in CD3+T cells and DNT cells in ER patients (Figure S5).

**Single cell RNA sequencing and data analysis**

Single-cell RNA sequencing was performed on BM aspirates from two ER and two CR patients (clinical characteristics in Table S2) using the 10x Genomics Chromium platform, targeting 10,000 cells per sample. Sequencing was conducted on an Illumina NovaSeq 6000 system, yielding an average of 340 million read pairs per sample. Reads were aligned to the GRCh38 reference genome using Cell Ranger (v7.2.0, 10x Genomics), with cell barcodes filtered to retain approximately 96% of cells. Data processing was performed by Microgen (Daejeon, Korea).

Data normalization, quality control, and clustering were conducted using Seurat (v5.0.1). Cells with >25% mitochondrial gene content, fewer than 200, or more than 7500 expressed genes were excluded. Clustering was performed using the Louvain algorithm and visualized via UMAP. Immune and hematopoietic cell types were manually annotated based on established markers. T cell subclusters were filtered using CD3 complex markers (CD3δ, CD3ε, and CD3ζ), and DNT cells were further identified using IKZF2, CD160, and ZBTB16. Differential gene expression analysis was conducted to assess T cell status and activation. Gene set enrichment analysis (GSEA) was performed using ssGSEA (v1.2.4, RRID:SCR_006442), and protein-protein interaction and pathway analyses were conducted using STRING (v12.0, RRID:SCR_005223) to explore T cell exhaustion and metabolic reprogramming.

**Cytotoxicity Assay**

The THP-1 cell line (TIB-202; RRID:CVCL_0006; ATCC, Manassas, VA, USA) was cultured in RPMI 1640 medium supplemented with 10% FBS and 1% penicillin/streptomycin in a 21% O₂ and 5% CO₂ atmosphere at 37°C for seven days before the assay. BM-derived lymphocytes were isolated from six patients (three CR and three ER), incubated under the same conditions for 8 hours, and CD3+ T cells were purified using the EasySep™ Human CD3 Positive Selection Kit II (100-0692, StemCell Technologies, Canada). Purified CD3+ T cells (50,000) were co-cultured with THP-1 cells (50,000) in the presence of anti-human CD3 monoclonal antibody (Mabtech, 3605-1-50) and sabatolimab at concentrations of 12.5, 25, and 50 μg/mL (280966, MedChemExpress, USA). After 24 hours, the cells were harvested, stained with CD45, incubated for 30 minutes at 4°C, and washed with PBS containing 20% BSA and 0.1% sodium azide. Apoptotic cells were identified using the FITC Annexin V Apoptosis Detection Kit with Propidium Iodide (640914, BioLegend, USA).

**Statistical analysis**

Categorical variables were compared using the chi-square test with SPSS version 24.0 (IBM, Armonk, NY, USA). Experimental data were analyzed using unpaired or paired two-tailed t-tests or one-way/two-way ANOVA followed by Dunnett’s post hoc analysis using GraphPad Prism v7.02 (GraphPad Software, La Jolla, CA, USA). Statistical significance was defined as *p* < 0.05. Significance levels are indicated as follows: *p < 0.05, **p < 0.01, ***p < 0.001, and ****p < 0.0001.
