# Supplementary material for: Elevated TIM3 expression on bone marrow T cells drives immune dysfunction in early relapsed blood cancer after allogeneic hematopoietic stem cell transplantation
Source: Exp Hematol Oncol. 2025 Aug 14;14:107. doi: 10.1186/s40164-025-00697-6 (PMC12355862; doi:10.1186/s40164-025-00697-6)
Supplement: Supplementary file 2 — Supplementary Material 2 [file 40164_2025_697_MOESM2_ESM.docx]

**Table 1**. Clinical characteristics between complete remission (CR) and early relapsed (ER) groups after HSCT (n=74).

|  | CR (n=55) | ER (n=19) | *p*-value |
| --- | --- | --- | --- |
| Median Age, year (range) | 57, (18-72) | 59, (23-70) | 0.831 |
| Gender, M:F | 27 : 28 | 6 : 13 | 0.284 |
| Type of diseases |  |  | 0.296 |
| AML | 28 (50.9%) | 14 (73.7%) |  |
| ALL | 12 (21.8%) | 2 (10.5%) |  |
| MDS & PMF | 15 (27.3%) | 3 (15.8%) |  |
| Type of donors |  |  | 0.497 |
| HLA-matched sibling | 22 (40.0%) | 8 (42.1%) |  |
| HLA-matched unrelated | 16 (29.1%) | 3 (15.8%) |  |
| Haplo-identical | 17 (30.9%) | 8 (42.1%) |  |
| Disease status at transplant |  |  | 0.167 |
| 1^st^ CR | 39 (70.9%) | 13 (68.4%) |  |
| 2^nd^ CR | 1 (1.8%) | 2 (10.5%) |  |
| 3^rd^ CR  Persistent | 1 (1.8%)  0 (0.0%) | 0 (0.0%)  1 (5.3%) |  |
| MDS & PMF | 14 (25.5%) | 3 (15.8%) |  |
| Poor risk* | 29 (70.7%) | 11 (64.7%) | 0.241 |
| HCT-CI |  |  | 0.479 |
| 0 | 29 (52.7%) | 12 (63.2%) |  |
| 1-2 | 20 (36.4%) | 4 (21.1%) |  |
| 3- | 6 (10.9%) | 3 (15.8%) |  |
| CMV reactivation | 12 (21.8%) | 3 (15.8%) | 0.746 |
| Acute GVHD (evaluable) at the time of BM sampling |  |  | 0.275 |
| None | 33 (60.0%) | 13 (68.4%) |  |
| Grade I/II | 20 (36.4%) | 4 (21.1%) |  |
| Grade III/IV | 2 (3.6%) | 2 (10.5%) |  |
| Stem cell source |  |  | - |
| PB | 55 (100%) | 19 (100%) |  |
| BM | 0 (0.0%) | 0 (0.0%) |  |
| Conditioning regimen |  |  | 0.430 |
| MAC | 26 (47.3%) | 7 (36.8%) |  |
| RIC | 29 (52.7%) | 12 (63.2%) |  |
| Cell count, median (range) |  |  |  |
| TNC count (x10^8^ cells/kg) | 14.21 (6.41-30.70) | 12.67 (8.90-24.47) | 0.328 |
| CD34+ cell (x10^6^cells/kg) | 6.57 (1.98-28.96) | 8.70 (2.60-20.78) | 0.489 |
| Time point sampling (days after HSCT), median (range) | 94 (74-110) | 118 (60-150) | 0.114 |
| Median F/U duration, month (range) | 13.5 (2.0-23.3) | 7.5 (2.7-15.2) | <0.001 |

*Poor risk includes sAML, tAML, AML with poor risk group in NCCN guideline, poor cytogenetics in ALL.

CR, complete remission; ER, early relapsed; HSCT, hematopoietic stem cell transplantation; HCT-CI, Hematopoietic stem cell transplantation comorbidity index; CMV, cytomegalovirus; PB, peripheral blood; BM, bone marrow; MAC, myeloablating conditioning; RIC, reduced intensity conditioning; TNC, total nucleated cell.
