# Supplementary material for: Elevated TIM3 expression on bone marrow T cells drives immune dysfunction in early relapsed blood cancer after allogeneic hematopoietic stem cell transplantation
Source: Exp Hematol Oncol. 2025 Aug 14;14:107. doi: 10.1186/s40164-025-00697-6 (PMC12355862; doi:10.1186/s40164-025-00697-6)
Supplement: Supplementary file 3 — Supplementary Material 3 [file 40164_2025_697_MOESM3_ESM.docx]

**Supplementary table 2.** Clinical characteristics of four patients who underwent single-cell RNA sequencing.

|  | | Complete remission | | Early relapse | |
| --- | --- | --- | --- | --- | --- |
| Age/gender | | 65/F | 53/M | 62/F | 46/F |
| Diagnosis | | MDS | ALL | AML | AML |
| At diagnosis | BM blast | 0.4 | 71.1 | 56 | 59.2 |
|  | Karyotype | t(1;20)(p34.1;q11.2) | t(9;22)(q34.1;q11.2) | Normal | Normal |
|  | Somatic mutation | Non-specific | PAX5 | NPM1, FLT3-ITD, DNMT3A, IKZF1 | FLT3-ITD, TET2 |
| At sampling time | BM blast | 0.5 | 0.3 | 0.9 | 14.1 |
|  | Karyotype | Normal | Normal | Normal | Normal |
|  | Somatic mutation | Not evaluated | Not evaluated | NPM1, FLT3-ITD | NPM1, FLT3-ITD |
| HSCT regimen | Conditioning | Reduced  intensity | Myeloablating | Reduced intensity | Reduced  intensity |
|  | Donor | Matched sibling | Matched sibling | Matched unrelated | Matched sibling |
|  | GVHD prophylaxis | ATG + cyclosporine | ATG + cyclosporine | ATG + tacrolimus | ATG + cyclosporine |
