# Supplementary material for: Elevated TIM3 expression on bone marrow T cells drives immune dysfunction in early relapsed blood cancer after allogeneic hematopoietic stem cell transplantation
Source: Exp Hematol Oncol. 2025 Aug 14;14:107. doi: 10.1186/s40164-025-00697-6 (PMC12355862; doi:10.1186/s40164-025-00697-6)
Supplement: Supplementary file 4 — Supplementary Material 4 [file 40164_2025_697_MOESM4_ESM.docx]

**Supplementary Table 3.** List of fluorochrome-conjugated monoclonal antibodies utilized in the present study.

| **Antibodies** | **Clone** | **Vendor** | **Catalogue number** | **Dilution** |
| --- | --- | --- | --- | --- |
| CD3-FITC | UCHT1 | Biolegend | 300406 | 1:25 |
| CD4-PerCP-Cy5.5 | OKT4 | Biolegend | 317428 | 1:50 |
| CD8-BV785 | RPA-T8 | Biolegend | 301046 | 1:100 |
| CD45RA-PE-Cy7 | HI100 | BD | 560675 | 1:50 |
| CCR7-PE-CF594 | 150503 | BD | 562381 | 1:25 |
| CD127-APC | A019D5 | Biolegend | 351316 | 1:34 |
| CD95-BV421 | DX2 | Biolegend | 305624 | 1:25 |
| CD25-BV421 | M-A251 | BD | 562442 | 1:34 |
| CD19-BV510 | HIB19 | Biolegend | 302242 | 1:67 |
| LAG3-PE | 11C3C65 | Biolegend | 369306 | 1:20 |
| TIGIT-Alex flour700 | MBSA43 | ThermoFisher | 56-9500-42 | 1:50 |
| TIM3-APC-Cy7 | F38-2E2 | Biolegend | 345025 | 1:20 |
| CTLA4-BV605 | BNI3 | Biolegend | 369610 | 1:34 |
| PD1-BV650 | EH12.2H7 | Biolegend | 329950 | 1:25 |
| TCRɣδ-PE | B1.1 | ThermoFisher | 12-9959-42 | 1:20 |
| TCRαβ-BV711 | IP26 | Biolegend | 306740 | 1:50 |
| CD16-PE-CF594 | 3G8(RUO) | BD | 562293 | 1:100 |
| CD161-PE-Cy7 | HP-3G10 | Biolegend | 339918 | 1:20 |
| CD56-BV421 | HCD56 | Biolegend | 318328 | 1:34 |
| DNAM1-BV605 | 11A8 | Biolegend | 338324 | 1:50 |
