# Supplementary material for: Elevated TIM3 expression on bone marrow T cells drives immune dysfunction in early relapsed blood cancer after allogeneic hematopoietic stem cell transplantation
Source: Exp Hematol Oncol. 2025 Aug 14;14:107. doi: 10.1186/s40164-025-00697-6 (PMC12355862; doi:10.1186/s40164-025-00697-6)
Supplement: Supplementary file 16 — Supplementary Material 16 [file 40164_2025_697_MOESM16_ESM.docx]

**Supplementary Figure Legends**

**Supplementary Figure 1.** Multiparameter flow cytometry analysis utilizing a gate approach on a sequence of bone marrow T cell surface markers, including T cell subpopulations and T cell inhibitor receptors*.* Strategy gating was performed for the analyses illustrated in Figures1A-1F, as well as in the supplementary figures 2, 3 and 4, which encompass all the different flow cytometry panels utilized.

**Supplementary Figure 2.** The expression level of the inhibitory receptors (IR) on the BM CD4^+^T and CD8^+^T cells in the control (N=9), CR (N=55) and ER (N=19) groups. **A** Percentages of IR-positive cells in CD4+T cells. **B** Percentages of IR-positive cells in CD8^+^T cells.

**Supplementary Figure 3.** The expression levels of inhibitor receptors on CD4^+^T cell and CD8^+^T cell differentiation subsets in the control (N=9), CR (N=55) and ER (N=19) groups. **A-B**, Naïve cells (CD45RA^+^CCR7^+^CD95^-^, CD8/CD4_Naïve_). **C-D**, Stem cell memory cells (CD45RA^+^CCR7^+^CD95^+^, CD8/CD4_SCM_). **E-F** show central memory cells (CD45RA^−^CCR7^+^, CD8/CD4_CM_). **G-H** are effector memory cells (CD45RA^−^CCR7^−^, CD8/CD4_EM_) T cells. **I-J,** Terminally differentiated effector memory cells (CD45RA^+^CCR7^−^, CD8/CD4_EMRA_). *Note: For each subset, samples with low cell numbers (<50–100 events) were excluded from MFI analysis to ensure statistical reliability and reduce noise. As a result, the number of samples may vary for each subset and marker, particularly in cases where expression was undetectable or very low. The majority of MFI results in certain subsets are negative or near-background, and only samples meeting the minimum cell count were included in statistical comparisons. According to two-way ANOVA multiple comparisons, statistically significant differences between the Normal, CR, and ER groups are indicated in black: *, p<0.05; **, p<0.01; ***, p<0.001.

**Supplementary Figure 4.** The percentage of cells expressing CD95 (Fas/apoptosis antigen 1), utilized as an apoptosis marker, was employed to estimate the process of cell death in the following cell types: CD3^+^T cell **(A),** CD4^+^T cell **(B),** CD8^+^T cell **(C),** DNT cell **(D)** in the control (N=9), CR (N=55) and ER (N=19) groups. The significance levels were determined using ordinary one−way ANOVA followed by multiple comparisons, with * indicating p<0.05, **p<0.01, and ****p<0.0001.

**Supplementary Figure 5.** A multiparameter flow cytometry gating approach was applied to analyze bone marrow DNT cell surface markers (15 CR patients and 6 ER patients). **A** subpopulation and inhibitory receptors of DNT cells. **B** the assessment of DNT cells’ capability to target cancer cells via the activation marker DNAM-1. Strategy gating was conducted for the analyses shown in Figure 1H-1J, covering all the different flow cytometry panels utilized.

**Supplementary Figure 6.** Percentage of DNT-TCRαβ, DNT-TCRɣδ and DNT-NKT between the CR (N=15) and ER (N=6) groups on total CD3^+^T cells.

**Supplementary Figure 7.** A multiparameter flow cytometry gating approach was applied to analyze bone marrow Tregs surface markers (54 CR patients and 19 ER patients). Gating Strategy was performed for the analyses shown in Figure 1L and 1M, encompassing all the distinct flow cytometry panels utilized.

**Supplementary Figure 8.** Transcriptional landscape of T-cell subsets four patients (two CR and two ER). **A** The T-cell subsets were selected by subclusters (CD3ε, CD3δ and CD3ζ, log2FC >1) into three main clusters: DNT, CD8^+^T cell and CD4^+^T cell. **B** A heatmap was utilized to accentuate the differentially expressed markers in each cluster (DNT, CD8+ and CD4+T cells) for the purpose of identifying key markers. The classified clusters manifest distinct and well-defined phenotypes, consistent with the established characteristics of classical T-cell analysis. **C, D** Expression of CD4 and CD8A Gene Groups: The expression of CD4 and CD8A gene groups, which differentiate T-cell types, accurately reflects CD4 and CD8 expression in both CR and ER samples. In contrast, the lower-left clusters, defined as DNTs, showed poor expression of both these markers, as expected. **E** A dot plot displaying major markers, including those for DNTs, illustrates distinct transcriptional profiles for CD4, CD8, and DNT-specific markers (*IKZF2, CD160*). This plot further revealed lower cytotoxic potential and TCR expression in ER samples compared to CR samples, particularly within the DNT population.

**Supplementary Figure 9.** The expression levels of inhibitory receptors were subsequently measured via flow cytometry in CD3^+^T cell **(A)** and DNT cells **(B)** of four patients, corresponding to the samples utilized for scRNAseq analysis.

**Supplementary Figure 10.** Expression levels of inhibitory receptors on each BM T–cell subtype was compared according to disease types (AML and non-AML). They were then further stratified based on donor origin into HLA-matched HSCT and HLA-haploidentical HSCT cohorts (AML; N= 42/ non-AML; N=32). The proportion of IR-positive cells on CD3^+^T cells **(A-B)**, CD4^+^T cells **(C-D)**, CD8^+^T cells **(E-F)**, and DNT cells **(G-H)** are shown according to disease type. Statistically significant differences between the Normal, CR, and ER groups are indicated in black: *, p<0.05; **, p<0.01; ***, p<0.001; ***, p<0.0001, as determined by two-way ANOVA multiple comparisons.

**Supplementary Figure 11.** Transcriptional landscape of myeloid-derived suppressor cells (MDSCs) in patients after allo-HSCT. **A** tSNE plot (top) showing the unsupervised clustering of MDSCs from four patients (two CR, two ER), with each dot representing a single cell colored by its cluster identity. Bar plots (bottom left) illustrate the fraction of cells derived from CR (cyan) and ER (orange) patients within each cluster. Notably, some clusters, particularly Cluster 2 (C2), Cluster 5 (C5), and Cluster 6 (C6), were found to be highly dominant in ER patients, indicating a shift in MDSCs composition. The total number of cells per cluster (bottom right) is also shown. **B** Feature plots of key MDSCs Markers. tSNE plots display the expression levels of canonical MDSCs-defining markers: *ITGAM* (CD11b), *CD14*, *FUT4* (CD15), and *HLA-DRA*. High expression is indicated by darker blue. Specifically, Cluster 2 is inferred to be of the polymorphonuclear MDSCs (PMN-MDSCs) phenotype due to its high expression of *FUT4*. These plots highlight distinct expression patterns that aid in the annotation of MDSCs subsets. **C** Cluster-Specific Differential Gene Expression of MDSCs-Associated Molecules. Violin plots show the expression levels of selected genes across the identified MDSCs clusters (0-10) for both CR (cyan) and ER (orange) samples. These plots illustrate increased expression of canonical MDSCs markers (*S100A8/S100A9*) and molecules associated with immunosuppression and pro-tumorigenic activity (*ARG1*, *VEGFA*, and *MMP9*) in ER patients. **D** Metabolic reprogramming pathways in MDSCs: pentose phosphate pathway (PPP) activation. A dot plot illustrating the gene set enrichment analysis (GSEA) results for the PPP, a key metabolic reprogramming pathway involved in the immunoregulatory profile of MDSCs. PPP activation was predominantly observed in the expanded cluster 2 (PMN-MDSCs) from ER patients (left), suggesting a metabolic shift contributing to their immunosuppressive characteristics. Bar plots on the right summarize the top enriched KEGG pathways in PMN-MDSCs from ER (enriched in ER, bottom right) versus CR (enriched in CR, top right), calculated using normalized enrichment scores (NES), further highlighting differentially regulated metabolic and functional pathways.
